# Arsenic alters nitric oxide signaling similar to autism spectrum disorder and Alzheimer’s disease-associated mutations

**DOI:** 10.1038/s41398-022-01890-5

**Published:** 2022-03-28

**Authors:** Manish Kumar Tripathi, Maryam Kartawy, Shelly Ginzburg, Haitham Amal

**Affiliations:** Institute for Drug Research, School of Pharmacy, Faculty of Medicine, The Hebrew University of Jerusalem, Jerusalem, Israel

**Keywords:** Molecular neuroscience, Learning and memory

## Abstract

Epidemiological studies have proven that exposure to Arsenic (AS) leads to the development of many neurological disorders. However, few studies have investigated its molecular mechanisms in the brain. Our previous work has revealed nitric oxide (NO)-mediated apoptosis and SNO reprogramming in the cortex following arsenic treatment, yet the role of NO and S-nitrosylation (SNO) in AS-mediated neurotoxicity has not been investigated. Therefore, we have conducted a multidisciplinary in-vivo study in mice with two different doses of Sodium Arsenite (SA) (0.1 ppm and 1 ppm) in drinking water. We used the novel SNOTRAP-based mass spectrometry method followed by the bioinformatics analysis, Western blot validation, and five different behavioral tests. Bioinformatics analysis of SA-treated mice showed significant SNO-enrichment of processes involved in mitochondrial respiratory function, endogenous antioxidant systems, transcriptional regulation, cytoskeleton maintenance, and regulation of apoptosis. Western blotting showed increased levels of cleaved PARP-1 and cleaved caspase-3 in SA-treated mice consistent with SA-induced apoptosis. Behavioral studies showed significant cognitive dysfunctions similar to those of Autism spectrum disorder (ASD) and Alzheimer’s disease (AD). A comparative analysis of the SNO-proteome of SA-treated mice with two transgenic mouse strains, models of ASD and AD, showed molecular convergence of SA environmental neurotoxicity and the genetic mutations causing ASD and AD. This is the first study to show the effects of AS on SNO-signaling in the striatum and hippocampus and its effects on behavioral characteristics. Finally, further investigation of the NO-dependent mechanisms of AS-mediated neurotoxicity may reveal new drug targets for its prevention.

## Introduction

Arsenic (AS) is a metalloid that is widely distributed in the air, water, and land [1]. New studies suggest the presence of AS in baby food and rice. The level of arsenic present in rice is mainly dependent on the country of origin and species of rice [2]. It ranges from 0.11 mg/kg to 0.2 mg/kg while the maximum accepted level of AS in rice-related products is 0.1 mg/kg according to the European Union [3]. Different processing or cooking methods of rice can reduce the AS contamination [2, 3]. It has also been used in medicine to treat syphilis, yaws, and amoebic dysentery [4], and recently to treat leukemia [5]. People can be exposed to a substantial level of inorganic AS by drinking contaminated water, manufacturing processes, eating rice-based food products, and smoking tobacco [6, 7]. AS can cause severe brain damage, and it is responsible for cognitive defects in children [8] in different countries such as Bangladesh [9, 10], Mexico [11, 12], and West Bengal, India [13].

The effects of AS on the nervous system have not received as much attention as its association with cancer, genotoxicity, and cellular disruption [14]. The toxic effects of AS on the nervous system can be manifested in encephalopathy [15, 16], peripheral neuropathy involving sensory and motor neurons [17, 18], sensory effects including ascending weakness and paralysis (in severe poisoning) [19], and other brain disorders. Oxidative stress and overproduction of reactive oxygen species (ROS), such as superoxide, hydroxyl radical, and hydrogen peroxide (H_2_O_2_), play an essential role in AS-induced brain damage [20–22]. Exposure to AS also suppresses the antioxidant defense system like glutathione and cytochrome C systems, superoxide dismutase (SOD), and catalase, thus exacerbating oxidative damage to DNA (deoxyribonucleic acid), proteins, and lipids [23]. AS-induced ROS production is largely driven by mitochondrial dysfunction [20]. Mitochondrial respiratory chain complexes I and III can contain electrons derived from NADH and ubiquinone that may interact directly with oxygen-generating ROS [24, 25]. Another primary source of mitochondrial ROS can be the loss of cytochrome C by mitochondria [26]. Oxidative damage to polyunsaturated fatty acids by lipid peroxidation has been widely accepted as a general mechanism for the toxic effects of AS [27]. AS-induced lipid peroxidation and generation of ROS have been suggested to be the leading cause of genotoxicity in laboratory animals [28, 29].

Along with oxidative stress, nitrosative stress represents another important cause of brain damage. Nitric oxide (NO) and H_2_O_2_ reciprocally enhance the production of each other [30]. Superoxide can rapidly interact with NO, leading to peroxynitrite production that breaks down DNA, lipid, and protein during oxidative stress [31, 32]. Other NO-related cell damage processes can be associated with proteins S-nitrosylation (SNO), tyrosine nitration, and S-nitrosoglutathione (GSNO) formation [33–36]. SNO represents the post-translational modification, in which a nitroso group is incorporated into a reactive cysteine thiol of the protein and forms a nitroso-thiol group [37, 38]. Under physiological conditions, SNO participates in protein localization, axonal transport, maintenance of synaptic plasticity, and regulation of various neuronal pathways [14, 23]. Recently, we have determined the SNO-proteins and found that there are significant differences in the brain between WT male and female mice [39], young and aged mice [40], and between different brain regions such as the striatum, cortex, and hippocampus [41]. These studies point to the importance of S-nitrosylation in maintaining normal cellular homeostasis and function. However, an increase in NO and SNO signaling may cause severe damage to the brain. We and others have shown its involvement in Autism Spectrum Disorder (ASD) [42], Alzheimer’s Disease (AD) [43, 44], Parkinson’s disease (PD) [45], and in other psychiatric and neurological disorders [46]. Importantly, we have recently reported that a low dose of AS in drinking water leads to a reprogramming of SNO in the cortex of mice. AS led to a Ca^2+^ release in cortical neurons and activation of NO-mediated apoptosis in cultured primary neurons [47].

**Fig. 1:**
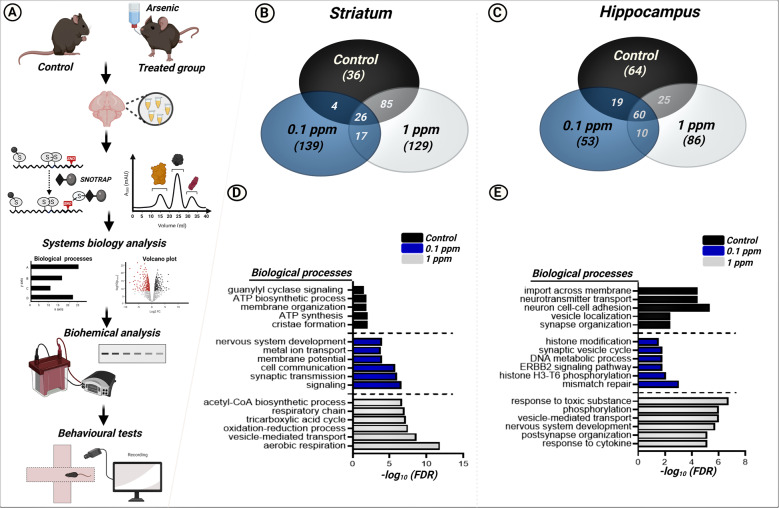
Systems biology analysis of the SNO-proteome. **A** Schematic workflow for the SNOTRAP-based mass spectrometry analysis of control and SA-treated mice groups followed by large-scale systems analysis, bioinformatics, biochemical, and behavioral studies**. B** Venn Diagram representing the striatal SNO-proteins identified in control and SA-treated groups**. C** Venn Diagram representing the hippocampal SNO-proteins identified in control and SA-treated groups**. D** BP analysis, conducted on the striatal SNO proteins that are exclusive to each of the control and both SA-treated groups**. E** BP analysis, conducted on the hippocampal SNO proteins that are exclusive to each of the control and both SA-treated groups. * Bars represent the –log10 of the Benjamini corrected false discovery rate (FDR).

The role of SNO in AS-mediated neurotoxicity, however, has not been thoroughly investigated. In this study, bioinformatics analysis suggested the involvement of SNO in sodium arsenite (SA)-induced damage to mitochondrial respiration and synaptic vesicle transport in the hippocampus and striatum regions accompanied by impairments of endogenous antioxidant systems, transcriptional regulation, cytoskeleton maintenance, and regulation of apoptosis. SA also caused behavioral deficits similar to those observed in ASD and AD mouse models. Recently, we compared between both ASD and AD mouse models and showed shared molecular mechanisms [48]. Finally, a comparative analysis of the SNO-proteome revealed similarities in the modulation of biological processes observed in SA-treated mice and the two different mutations that lead to ASD and AD pathologies. Figure 1A describes the flowchart of our current study.

## Material and methods

### Materials

SNOTRAP was purchased from Spirochem AG (Basel, Switzerland). All primary and HRP conjugated secondary antibodies were purchased from Cell Signaling Technology (Danvers, MA, USA). Other general chemicals were purchased from Sigma Aldrich (St. Louis, MO, USA) and Bio-Rad Laboratories (Haifa, Israel).

### Animal housing and tissue dissection

The guidelines of the Institutional Animal Care Committee of The Hebrew University (Jerusalem, Israel) were followed in this study. The study protocols have been approved by the joint ethics committee (IACUC) of The Hebrew University (AAALAC internationally accredited institution) and Hadassah Medical Centre. Juvenile (6–8-week-old) male C57BL/6NTac (Taconic Laboratories) mice were used in this study. The animals were kept at room temperature (RT) of 23 °C in a 12-hour light/dark cycle and fed ad libitum with standard mouse chow and water. SA was added to drinking water ad libitum for one month at two different doses (0.1 ppm and 1 ppm). Mice of the control group drank SA-free water. After 1 month, mice were sacrificed, and tissues were collected as described previously [42]. Briefly, the entire striatum and hippocampus were isolated and transferred to liquid nitrogen for storage at −80 °C. The two regions were studied for their well-established role in several neurological disorders [49–53]. Mice were randomly selected for tissue dissection, and the samples were also prepared in a similar way. The ASD and AD mouse models used in this study are described elsewhere [42, 44]. The following five groups of mice were employed in this study: 0.1 ppm, 1 ppm, Shank3, tau P301S, and control wild-type (WT) C57BL/6NTac mice. For the behavioral and biochemical tests, 5 mice of WT and SA-treated group were used. For MS lysate preparation, for each group, 3 striatal and 3 hippocampal tissue samples from 3 different mice were pooled into each of the six replicates [44].

### Brain tissue homogenization and samples preparation for Mass Spectrometry and Western blot analysis

Tissues were homogenized in freshly prepared lysis buffer (250 mM HEPES-NaOH, pH 7.7, 0.1 mM neocuproine, 1 mM EDTA, 1% Triton x-100, 10 mM iodoacetamide (IAM), 1% protease inhibitors cocktail) on ice using Teflon pestle and a Jumbo Stirrer (Thermo Fisher Scientific, Waltham, MA, USA). The homogenates were centrifuged (10,000×*g* for 10 mins at 4 °C). The supernatant was collected, and protein concentration was estimated by Bicinchoninic Acid (BCA) Protein Assay. Negative controls were generated by treatment with 5 mM Tris(2-carboxyethyl) phosphine hydrochloride (TCEP) for 30 mins at 37 °C after sample mixing. Next, in the presence of 2.5% SDS, samples were alkylated with 10 mM IAM in the dark at 37 °C. After alkylation, samples were washed twice with 3 times volumes of 6 M Urea (in 50 mM HEPES, pH 7.7) and once with 50 mM HEPES (pH 7.7) by centrifugation (5000×*g* for 30 min at 4 °C) with 10 kDa molecular weight cut-off (MWCO) spin filters pre-rinsed, once with water (Sartorius AG, Göttingen, Germany). After centrifugation, SNOTRAP labeling stock solutions (in 40% acetonitrile (ACN)) were added to all samples to reach a final concentration of 1.5 mM (in 50 mM HEPES buffer at pH 7.7) to selectively convert SNO to stable disulfide-iminophosphorane. All samples were incubated at RT for 2 hrs in SNOTRAP solution. After SNOTRAP labeling, excess reagents were removed by three continuous bouts of washings with 50 mM HEPES (pH 7.7) buffer with 10 kDa MWCO spin filters. Each sample was incubated after ultrafiltration with 200 μl pre-rinsed Streptavidin agarose beads (Thermo Fisher Scientific, Waltham, MA, USA) for 1 hr at RT with gentle shaking. The beads were washed with washing buffer (50 mM HEPES, 150 mM NaCl, 0.05 % SDS, pH 7.7) three times, then with a washing buffer (50 mM HEPES, pH 7.7) three times. Proteins were eluted (with 10 mM TCEP in 50 mM HEPES, pH 7.7) after washing, and then alkylated with 10 mM iodoacetamide (IAM). Protein samples were then trypsinized (Promega, Madison, WI, USA) at 37 °C for 4 hrs and subsequently desalted with C18 StageTips as previously described [54]. For Western blot experiments, the striatal tissue was homogenized and sonicated in RIPA buffer (Sigma Aldrich, St Louise, MI, USA, Cat. No. R0278) containing a protease and phosphatase inhibitor cocktail, sonicated, centrifuged at 4 °C, and a supernatant was collected. Protein content was measured in the supernatant using BCA protein assay (Sigma Aldrich, St Louise, MO, USA).

### Materials and reagents for Mass Spectrometry

The materials and reagents for Mass Spectrometry (MS) were procured from the following companies: Biotin-PEG3-propionic acid (Chem Pep Inc, Wellington, FL, USA), Vivapsin 10 kDa MWCO spin filters (Sartorius AG, Göttingen, Germany), sequencing-grade modified trypsin (Promega, Madison, WI, USA), and protease inhibitors cocktail, ACN, distilled water (Sigma-Aldrich, St. Louis, USA). SNOTRAP-biotin synthesis and nuclear magnetic resonance analysis were performed as previously described [55]. All samples were prepared at room temperature in the dark.

### Mass Spectrometry analysis

Water with 0.1% FA was used as mobile phase A, and ACN with 0.1% formic acid (FA) was used as mobile phase B. Protein digests were analysed on an Agilent 6550 Nano-HPLC-Chip/MS system, coupled with a micro-autosampler, a capillary, and nanoflow pump, as well as the Chip-Cube that interfaces LC modules and the MS instrument. Peptides were loaded onto the enrichment column from the autosampler at a constant flow of 2.5 μL/min provided by the capillary pump. A 50-min gradient started at 3% B at 300 nL/min and increased to 35% B from 2 to 35 min, to 60% B at 40 min, to 90% B at 45 min, and then held for 2.5 min, followed by a 2.5 min post-run at 3% B. Positive-ion MS spectra were acquired in the 1,700 Da extended dynamic range mode (2 GHz) using the following setting: electrospray ionization capillary voltage, 1,800 V; fragmentor, 360 V; Octopole radio frequency peak, 750 V; drying gas, 13 L/min; drying temperature, 225 °C. Data were acquired at a rate of 6 MS spectra per second and 3 MS/MS spectra per second in the mass range of m/z 300–1,700 for MS and 50–1,700 for MS/MS, then stored in centroid mode. The maximum number of precursors per cycle was 20, with a threshold of 5,000 ions in a precursor abundance-based scan speed in peptide isotope model, with +2, +3, and above charge-state preference, and with active exclusion after 1 spectrum and released after 0.15 min. Fragmentation energy was applied at a slope of 3.1 V/100 Da with a 1.0 offset for doubly charged precursors, 3.6 V/100 Da with a −4.8 offset for triply and multiply charged precursors. Mass accuracy was maintained by using an internal reference ion *m/z* 1221.9906. Agilent MassHunter Workstation software was used for data acquisition. MS data processing was conducted as described in our previous study [42].

### Western blots

For Western blot (WB) assays we used a set of equipment from Bio-Rad Laboratories (Hercules, CA, USA). The protein content in the samples was estimated and then subjected to polyacrylamide gel electrophoresis followed by wet transfer onto a PVDF membrane. Non-specific sites were blocked by either 5% dried skimmed milk or 5% BSA in tris-buffered saline (135 mM NaCl, 2.5 mM KCl, 50 mM Tris, and 0.1% Tween 20, pH 7.4) for 2 h at room temperature. PVDF membranes containing transferred proteins were incubated with a primary antibody overnight at 4 °C in shaking conditions. The following primary antibodies (all from Cell Signaling Technology, Danvers, MA, USA) were used: anti-P-m-TOR (1:1000 dilution, Cat number #5536), anti-m-TOR (1:1000 dilution, Cat number #2983), anti-Beta Actin (1:1000 dilution, Cat number #3700), anti-cleaved PARP (1:2000, Cat number #94885), anti-cleaved caspase 3 (1:1000, Cat number #9661), anti-p62 (1:1000, #88588), anti LC3 (1:1000, Cell signaling technology Cat number #3868) for overnight at 4 °C. After exposure to primary antibodies, the membranes were washed with TBST buffer and incubated with anti-mouse/rabbit-horseradish peroxidase-conjugated secondary antibody for 1 hr at RT. Specific binding of the protein of interest was detected using ECL substrate (Bio-Rad Laboratories, Hercules, CA, USA). The bands were visualized with the Bio-Rad Chemidoc imaging system (Hercules, CA, USA).

### Behavioral tests analysis

#### Open field test

The motor activity of mice was tested in an open field consisting of a white plastic arena (60 cm × 60 cm) with a floor divided into 10 cm × 10 cm squares. In the first session (habituation) the mouse was introduced to the field for 5 min. On the next day, the mouse was placed in the same field, and the number of squares that the mouse crossed during a 5 min session was counted [56].

#### Object recognition test

This test, as described in Amal et al. [56], utilizes the tendency of mice to explore novel stimuli. The test consisted of two parts: a familiarization session and a test session. In all, 24 hrs before these sessions, the mice were allowed to explore the arena without objects for 5 min to habituate to their surroundings. During the first 5 min session, the mice were left to explore two identical objects that could be found at constant locations, 15 cm from the sidewalls, in the already familiar plastic arena. 24 hrs later, the mice were introduced to the arena for a test session in which one of the familiar objects was replaced with a novel object. Exploration was defined as directing the nose to the object at a distance of ≤1 cm and/or touching the object with the nose. The time spent by the mouse in exploring each object was recorded for 5 min. The arena and the objects were cleaned with alcohol after each session.

#### Novel object exploration test

As described in Tyler and Allan [8], the test began with a familiarization session for the mice to explore the apparatus for 5 min. After the first session, a novel object was placed in the center area. The time spent by the mouse in exploring the novel object was recorded for 5 min. Afterward, the arena and the objects were cleaned with alcohol after each session.

#### Elevated plus maze test

The elevated plus-maze consists of four arms (30 × 5 cm), two open, and the other two closed. The platform was made of white plexiglass. The apparatus was elevated 45 cm above the floor. The test, described in Walf and Frye [57], was initiated by placing the mouse on the central platform of the maze, facing one of the open arms, and letting it move freely. Each session lasted 5 min. The time spent in the close and open arms was recorded.

#### Three-chambered social test

A three-chamber social test was performed as described in Silverman et al. [58] with some modifications. The social test apparatus consisted of a transparent acrylic box divided into three chambers. Two cylindrical wire cages were placed, one in chamber 1 and the other in chamber 2. For the sociability test, the test animal was introduced to the middle chamber and allowed to adjust for 5 min. Then, an unfamiliar mouse was introduced into a wire cage in one of the side-chambers, and the other side-chamber remained empty. The time spent by the test mouse exploring the wire cage with an unfamiliar mouse inside was recorded for 5 min.

#### Statistics and bioinformatics

For analysis of functional enrichment of Cellular Compartments (CC), Biological Processes (BP), and Molecular Functions (MF), we uploaded the lists of all S-nitrosylated proteins into MetaCore (Thomson Reuter, MetaCore™ version 6.34 build 69200 software). A search tool for the retrieval of interacting Genes/Proteins (STRING, version 10.0) was used to analyse the protein-protein interaction of SNO-proteins (http://string-db.org) [59]. High confidence interactions (score > 0.7) from the neighborhood, gene fusion, co-occurrence, co-expression, experiments, databases, and text-mining lists were used. The quantification was based on the ion intensity of the peptides. We used Benjamini-Hochberg correction [60] on the *p*-value to generate False Discovery Rate (FDR), and processes/terms with FDR values below 0.05 were included. Cytoscape version 3.3.0 software was used for the visualization of protein-protein interaction. GraphPad PRISM 8 software was utilized to generate schematic figures, heatmaps, and perform the statistical analysis. Mean and Standard Error of the mean (SEM) were calculated for the Western blots and behavioral experiments. Unpaired two-tailed *t-*tests were conducted with *P*-values < 0.05.

## Results

To test the effects of SA on the SNO-proteome in the striatum and hippocampus in a dose-dependent manner, we profiled the SNO-proteome in the two SA-treated groups of mice, 0.1 ppm, 1 ppm, and in the non-treated control group using SNOTRAP-based mass spectrometry technology. This was followed by bioinformatics analysis. We also conducted a large-scale quantitative analysis of the shared S-nitrosylated proteins between the control vs 0.1 ppm group and the control vs 1 ppm group. Next, we investigated the protein level of cleaved PARP-1 and cleaved caspase-3 to assess apoptosis. Then, behavioral experiments were conducted to test whether exposure to SA can induce cognitive alterations. Finally, we carried out a comparative analysis of the SNO-proteome between SA-treated mice and two mouse models of ASD and AD.

### SA leads to an increase in the number of S-nitrosylated proteins in the hippocampus and striatum

We identified 436 S-nitrosylated proteins in the striatum region of the brain, in which 36 S-nitrosylated proteins are exclusive to the control group; 139 proteins were exclusive to the 0.1 ppm group; 129 proteins were identified only in the 1 ppm group. Along with this, we found 17 shared SNO proteins between 0.1 ppm and 1 ppm groups (Fig. 1B). A total of 317 S-nitrosylated proteins were also found in the hippocampus region, in which 64 belonged exclusively to the control group, 53 to the 0.1 ppm group, and 86 to the 1 ppm group. 10 S-nitrosylated proteins appeared to be shared between 0.1 ppm and 1 ppm groups (Fig. 1C). For the detailed lists of S-nitrosylated proteins in the striatum and hippocampus of all groups, see Supplementary Tables 1 and 2, respectively.

### Bioinformatics analysis of the SNO-proteome in the striatum

Large-scale bioinformatics analysis was performed to better understand the involvement of biological and functional processes of SNO proteins in the striatum following SA treatment. Gene ontology (GO) analysis, including BP, CC, and MF of both treated groups, showed significant enrichment of many processes that are mainly involved in neuronal, mitochondrial, and cellular respiratory functions.

In the control group, BP analysis revealed the enrichment of a receptor guanylyl cyclase signaling pathway (FDR = 0.0298), inner mitochondrial membrane organization (FDR = 0.0150), ATP biosynthetic process (FDR = 0.0145), cristae formation (FDR = 0.0093), and mitochondrial ATP synthesis-coupled proton transport (FDR = 0.0093) (Fig. 1D). MF analysis of the control group showed enrichment of interleukin-8 receptor binding (FDR-0.0011), small molecule binding (FDR = 0.0018), ATP binding (FDR = 0.0042), syntaxin-1 binding (FDR = 0.0114), and calcium-induced calcium release activity (FDR = 0.0350) (Supplementary Fig. S1A). CC analysis revealed that SNO proteins were functionally enriched in different cellular locations, such as smooth endoplasmic reticulum (FDR-0.0088), intracellular organelle (FDR = 0.0088), and cytoplasmic part (FDR = 0.0126) (Supplementary Fig. S1B). More details about GO analysis of the control striatal group are presented in Supplementary Table 3.

BP analysis of the 0.1 ppm group revealed the enrichment of regulation of metal ion transport (FDR = 0.0001), nervous system development (FDR = 0.0001), regulation of membrane potential (FDR = 0.0001), regulation of cell communication (FDR = 1.7548E-06), and modulation of chemical synaptic transmission (FDR = 8.84E-07) (Fig. 1D). MF analysis showed the enrichment of nitric oxide synthase regulator activity (FDR = 0.0030), calcium channel inhibitor activity (FDR = 0.0033), G protein-coupled glutamate receptor binding (FDR = 0.0043), nitric-oxide synthase binding (FDR = 0.0067), and chaperone binding (FDR = 0.0154) (Supplementary Fig. S1A). CC analysis showed the presence of SNO proteins in different cellular localizations such as intracellular organelle part (FDR = 3.078E-07), cell projection (FDR = 3.613E-07), and plasma membrane-bounded cell projection (FDR = 3.61E-07) (Supplementary Fig S1B). See Supplementary Table 4 for more details about GO analysis of 0.1 ppm, striatal group.

BP analysis of 1 ppm group showed enrichment of acetyl-CoA biosynthetic process (FDR = 2.1866E-07), respiratory electron transport chain (FDR = 9.80E-08), tricarboxylic acid cycle (FDR = 5.91E-08), oxidation-reduction process (FDR = 3.11E-08), vesicle-mediated transport (FDR = 2.36E-09), exocytosis (FDR = 1.28E-11), and aerobic respiration (FDR = 1.48E-12) (Fig. 1D). Key S-nitrosylated proteins that are present in these biological processes are citrate synthase, isocitrate dehydrogenase, and malate dehydrogenase. We have discussed their importance in the Discussion section. MF analysis reveals the enrichment of nucleoside-triphosphatase activity (FDR = 6.45E-09), protein binding (FDR = 1.34E-07), peroxiredoxin activity (FDR = 1.58E-05), cadherin binding (FDR = 1.58E-05), and ATPase activity (FDR = 3.78E-05) (Supplementary Fig. S1A). CC analysis showed the presence of SNO proteins in myelin sheath (FDR = 8.34E-31), mitochondrial part (FDR = 6.91E-14), mitochondrion (FDR = 1.04E-12), and cytoplasmic part (FDR = 7.078E-10) (Supplementary Fig. S1B). See Supplementary Table 5 for more details about GO analysis of 1 ppm striatal group.

### Bioinformatics analysis of the SNO-proteome in the hippocampus

BP analysis of the hippocampus of the Control group also demonstrated the enrichment of different processes involved in various signalings such as import across the plasma membrane (FDR = 3.7631E-05), neurotransmitter transport (3.6004E = 05), neuron cell–cell adhesion (FDR = 4.4649E-06), vesicle localization (FDR = 0.0038), and synapse organization (FDR = 0.0038) (see Fig. 1E). CC analysis showed the localization of S-nitrosylated proteins in neuron part (FDR = 6.38E-05), axon (FDR = 0.0007), synapse part (FDR = 0.0007), and presynapse (FDR = 0.0034) (Supplementary Fig. S1C). See Supplementary Table 6 for more details about GO analysis of this group.

BP analysis of 0.1 ppm group showed the functional enrichment of regulation of mismatch repair (FDR = 0.0008), histone H3-T6 phosphorylation (FDR = 0.0086), regulation of response to nutrient levels (FDR = 0.0131), ERBB2 signaling pathway (FDR = 0.01482), regulation of DNA metabolic process (FDR = 0.0148), regulation of synaptic vesicle cycle (FDR = 0.0152), and histone modification (FDR = 0.0299) (Fig. 1E). Key S-nitrosylated proteins that are present in these biological processes are histone lysine demethylase, N-terminal acetyltransferase 50, profilin, protein tyrosine kinase 7 (PTK7), spectrin, myelin basic protein, beta fodrin, actinin alpha 1, F-actin etc. We have discussed their importance in the Discussion section. CC analysis showed enrichment of S-nitrosylated proteins in retromer complex (FDR = 0.0052), cytosol (FDR = 0.0052), the extrinsic component of endosome membrane (FDR = 0.0293), and ubiquitin ligase complex (FDR = 0.0316) (Supplementary Fig. S1C). See Supplementary Table 7 for more details about GO analysis of the 0.1 ppm group.

BP analysis of 1 ppm group reveals the functional enrichment of response to cytokine (FDR = 7.15E-06), postsynapse organization (FDR = 7.15E-06), nervous system development (FDR = 1.74E-06), vesicle-mediated transport (FDR = 9.68E-07), phosphorylation (FDR = 9.37E-07), cellular response to toxic substance (FDR = 1.85E-07), and cytoskeleton organization (FDR = 2.62E-12) (Fig. 1E). CC analysis showed S-nitrosylated proteins in various cellular locations such as cytoskeleton (FDR = 1.32E-07), extracellular exosome (FDR = 1.48E-07), extracellular vesicle (FDR = 1.48E-07), and extracellular organelle (FDR = 1.48E-07) (Supplementary Fig. S1C). See Supplementary Table 8 for more details about GO analysis of the 1 ppm group.

### Protein–protein interaction network analysis of the SNO-proteome

Physical interaction analysis was conducted on the SNO proteins of the 1 ppm SA-treated group of mice both in the striatum and hippocampus. Following the exposure to 1 ppm of SA, the SNO proteins formed a distinct network in the striatum (Supplementary Fig. S2A) that showed the involvement of AS in several processes related to mitochondrial energy generation, such as aerobic respiration, oxidation-reduction process, acetyl-CoA biosynthetic process, respiratory electron transport chain, and TCA. In the hippocampus, the SNO proteins formed a network (Supplementary Fig. S2B) involved in the regulation of neuronal processes such as post-synapse organization, nervous system development, vesicle-mediated transport, and cellular defense systems (e.g., response to cytokine, toxic substances, and phosphorylation). Network analysis of the 0.1 ppm groups did not reveal significant clusters.

### Pathway analysis of the SNO proteins in striatum and hippocampus

We conducted pathway analysis to identify the affected pathological signaling pathways following 0.1 ppm and 1 ppm SA exposure. In the striatum, pathway analysis revealed significant enrichment of “Synaptic vesicle fusion and recycling in nerve terminals” pathway in the 1 ppm treated group (FDR = 0.0003743) but this pathway was not significantly SNO-enriched in the 0.1 ppm group (FDR = 0.1671), which emphasizes the dose-dependent effect of SA (Supplementary Fig. S3 and Supplementary Table 9). In the hippocampus of the 0.1 ppm and 1 ppm SA-treated groups, the pathway “mTORC2 downstream signaling” was SNO-enriched with FDR of 0.0439 and 0.0005, respectively (Supplementary Fig. S4 and Supplementary Table 10). See also Supplementary Tables 9 and 10 for the lists of enriched pathways in the SA-treated groups in the two brain regions.

### Quantitative analysis of SNO-proteome in striatum and hippocampus

**Fig. 2:**
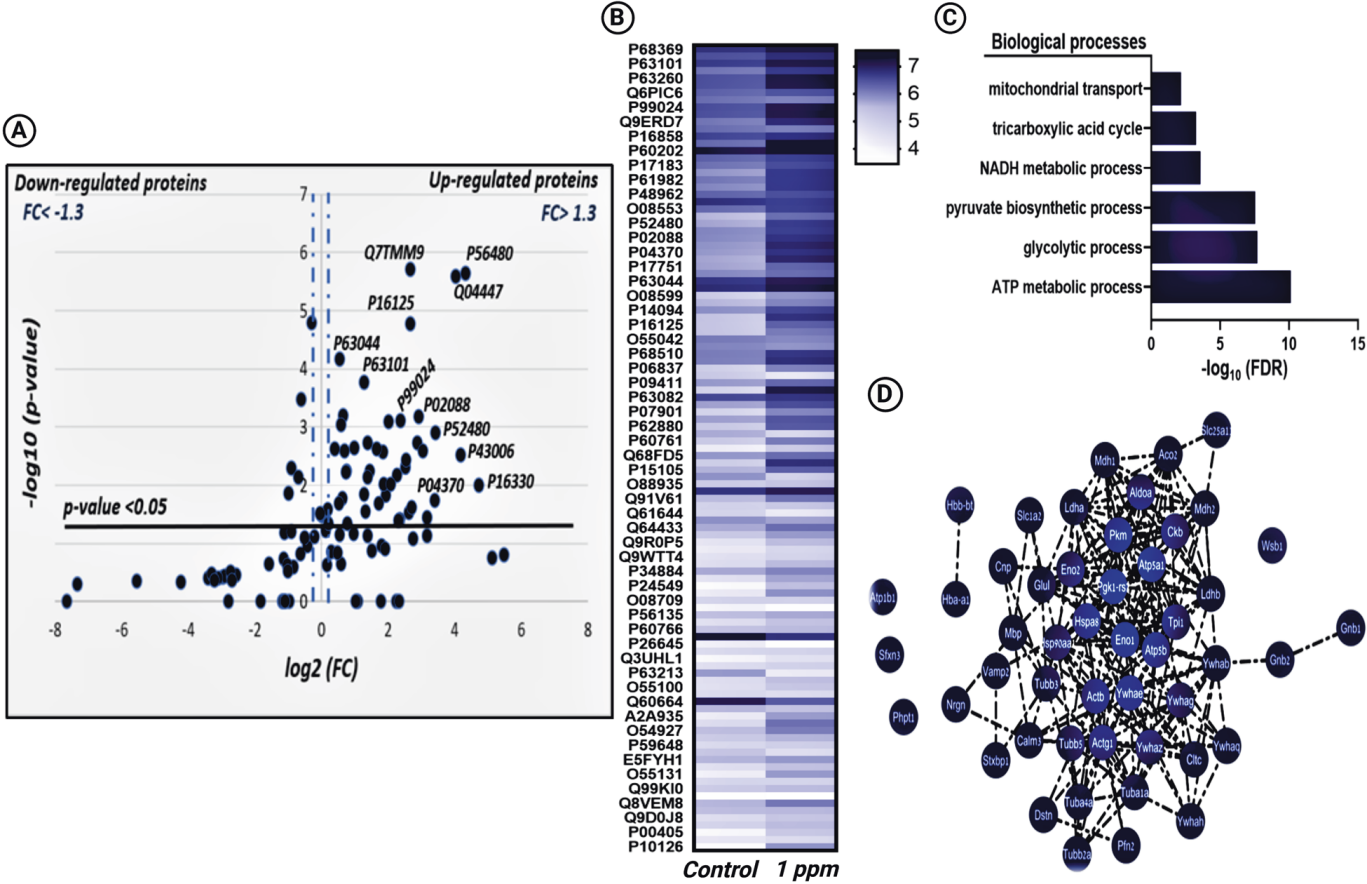
Quantitative analysis of the SNO-proteins in Striatum of 1 ppm group. **A** Volcano Plot analysis was conducted on the striatal shared SNO proteins between the control and 1 ppm group. The *X*-axis represents the fold change (log2(FC)) that was calculated as the difference in the relative abundance of each protein in both tested groups, divided by the relative abundance of the protein in control [FC = (Relative abundance(SA) − Relative abundance (control))/(relative abundance(WT))]. The *Y*-axis represents the −Log10 of the *p*-value. The black horizontal line represents a significance level of *P*-value = 0.05. The two fragmented vertical lines represent the threshold of the FC = 1.3. Proteins that are upregulated in the 1 ppm group appear on the right side of the plot with statistical significance of *p*-value < 0.05 and FC > 1.3**. B** Heat map analysis representing the differential relative abundance of the shared SNO-proteins in the control vs 1 ppm group. The relative abundance scale was normalized by –log10**. C** BP analysis conducted on the upregulated SNO proteins in the 1 ppm group as compared to the control group**. D** Clustering analysis of upregulated SNO proteins in the 1 ppm group as compared to the control group.

We conducted a large-scale quantitative analysis to understand and visualize the extent of changes that occur under the exposure to SA in a dose-dependent manner. We hypothesized that increasing the dose of SA leads to an increase in S-nitrosylation intensity. To visualize these quantitative differences, we built a Volcano plot and heat map of the SNO proteins in the striatum and hippocampus of untreated (control) and treated mice (0.1 ppm and 1 ppm) (Fig. 2 and Supplementary Figs. S5 and S6).

In the striatum, a total of 111 proteins shared between the control and the 1 ppm groups were tested. The analysis reveals a total of 50 proteins significantly more abundant in the 1 ppm group (Fold Change (FC) > 1.3 and *p* < 0.05; Fig. 2A). Heat maps visualize the differences in the relative abundance of the proteins shared between the control and 1 ppm groups (Fig. 2B) and between the control and 0.1 ppm groups of mice (Supplementary Fig. S5B). GO analysis of the upregulated proteins in the 1 ppm group showed that they are involved in the biological processes related to mitochondrial functioning. Thus, the following biological processes were found to be upregulated in this group: mitochondrial transport (FDR = 0.0070), TCA cycle (FDR = 0.0006), NADH metabolic processes (FDR = 0.0003), pyruvate biosynthetic processes (FDR = 2.81E-08), glycolytic processes (FDR = 1.91E-08), and ATP metabolic processes (FDR = 7.08E-11) (Fig. 2C). Figure 2D presents the clustering analysis of the upregulated proteins. 30 proteins appeared to be shared between the control and the 0.1 ppm group. Among them, 14 proteins were more abundant in 0.1 ppm of SA (Supplementary Fig. S5A). A small but significant difference was observed in the hippocampus between the shared proteins of the control and 1 ppm group (Supplementary Fig. S6A). The relative abundance of proteins shared between the control and 0.1 ppm group was slightly but significantly higher in the latter mice (Supplementary Fig. S6B).

### Western blot analysis of apoptotic and autophagic markers in striatum

**Fig. 3:**
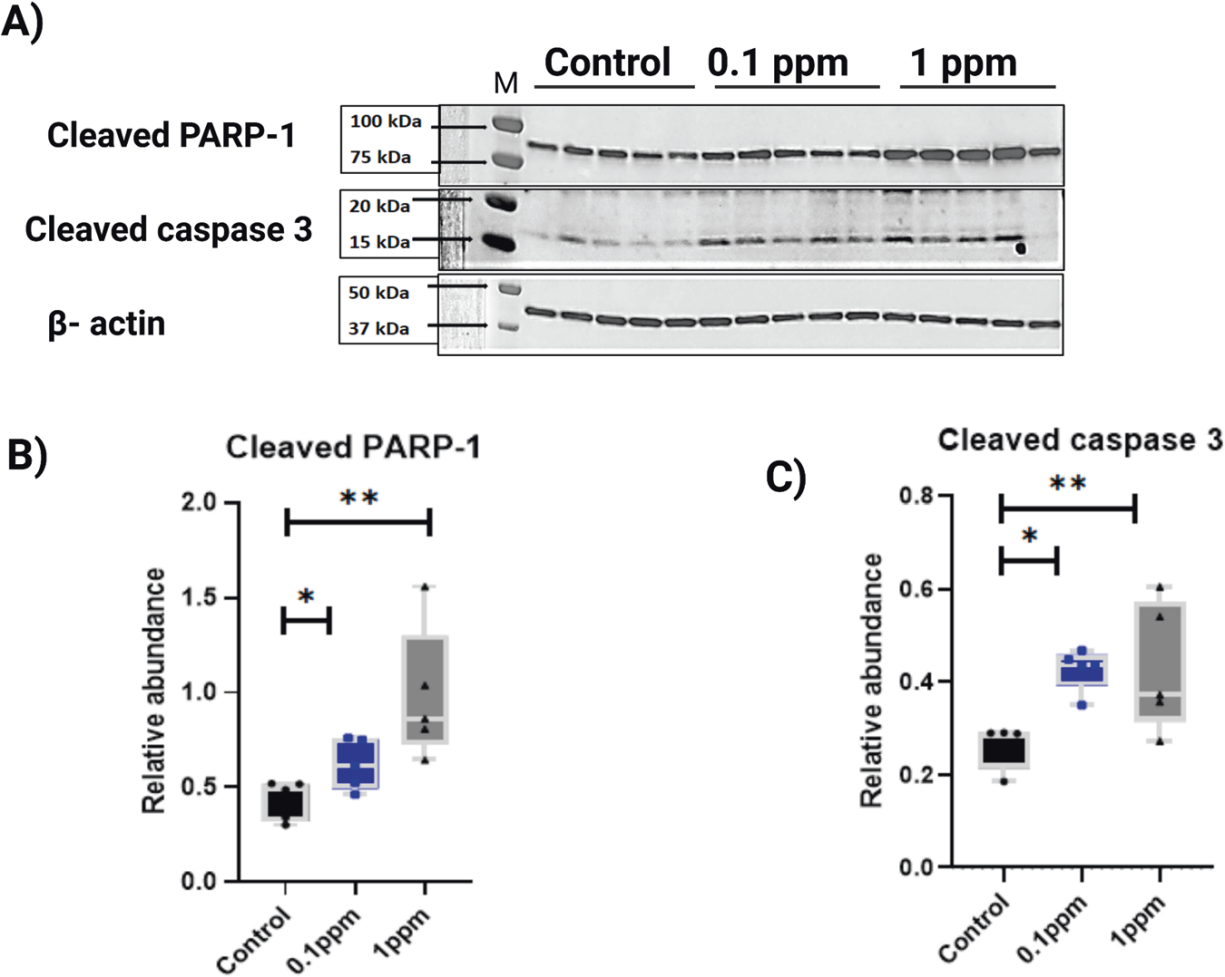
Western blot validation of the apoptotic biomarkers. **A** Representative Western blot of striatal homogenate from control, 0.1 ppm group and 1 ppm groups show a significant difference in the protein level of cleaved PARP 1 (molecular weight-89) and cleaved caspase 3 (molecular weight-17) in the striatum of 0.1 ppm and 1 ppm group as compared with the control group. The quantitative analysis is shown in **B** for cleaved PARP 1 and in **C** for cleaved caspase 3 in all groups. The data are presented as mean values ±SEM (*n* = 5). Two-tailed *t*-tests was conducted.

GO analysis showed significant SNO-enrichment of mitochondrial regulation processes. Meanwhile, it has been shown that excessive NO and SNO could be responsible for neuronal cell death [61]. We, therefore, conducted Western blot analysis of the marker proteins of apoptosis (cleaved PARP-1 and cleaved caspase 3; Fig. 3) and autophagy (p-62, LC3, and P-mTOR; Supplementary Fig. S7) in the brain of control and the two SA-treated groups of mice. Western blot analysis showed a higher protein level of cleaved PARP-1 in 0.1 ppm and 1 ppm SA-treated animals in the striatum region compared to the control group (Fig. 3B). Cleaved caspase3 was also increased in SA-treatment groups compared to control (see Fig. 3C). These findings indicate activation of apoptosis in the striatum region following exposure of mice to SA in drinking water. We investigated the protein level of autophagy biomarkers (LC3, p-62, and p-mTOR) in all groups, but no significant changes were found (see Supplementary Fig. S7).

### Effects of arsenic consumption on mouse behavior

**Fig. 4:**
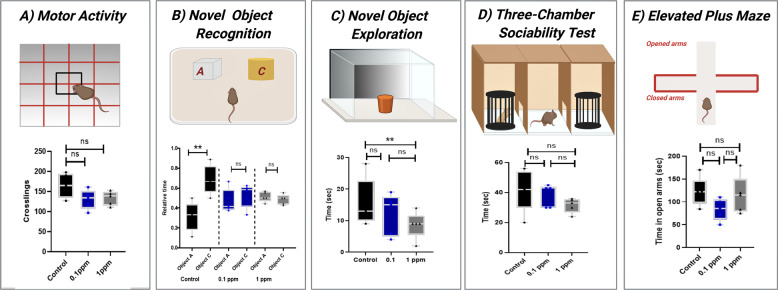
Behavioral tests to examine cognitive deficits. **A** Motor activity shows no significant differences between the groups. **B** In the novel object recognition test, the SA-treated mice failed to differentiate between the novel and previously encountered objects while the control group spent significantly more time exploring the new object. **C** In the novel object exploration test, the 1 ppm group spent significantly less time exploring a novel object than control mice. **D** In a three-chamber sociability test, there is no significant difference between the SA-treated groups and control mice. **E** An elevated plus-maze test showed no difference among the three groups.

To test whether the neurotoxic effects of low doses (0.1 and 1 ppm) of SA lead to cognitive behavioral dysfunctions, we conducted a large set of behavioral tests in the SA-treated and non-treated groups. Still, the link between AS, NO, and behavior need to be investigated in future studies. First, motor activity [56] was tested and no significant difference among the groups was found (Fig. 4A). Next, we tested the memory, learning, and tendency of the mice to explore novel stimuli. In the novel object recognition test (NOR, Fig. 4B), the SA-treated mice failed to differentiate between the novel and previously encountered objects, while the control group spent significantly longer time exploring the new object. To confirm this behavioral pathology, we also conducted a novel object exploration (NOE) test and found that mice of the 1 ppm group spent significantly shorter time exploring a novel object than the control mice (Fig. 4C). The three-chamber sociability test was conducted to determine abnormal social interaction [58]. We found no significant difference between the SA-treated groups and control mice (Fig. 4D). An elevated plus maze test used to investigate anxiety [57]. It also showed no difference among the three groups (Fig. 4E).

### Comparative bioinformatics analysis between SA-treated mice and ASD and AD mouse models

**Fig. 5:**
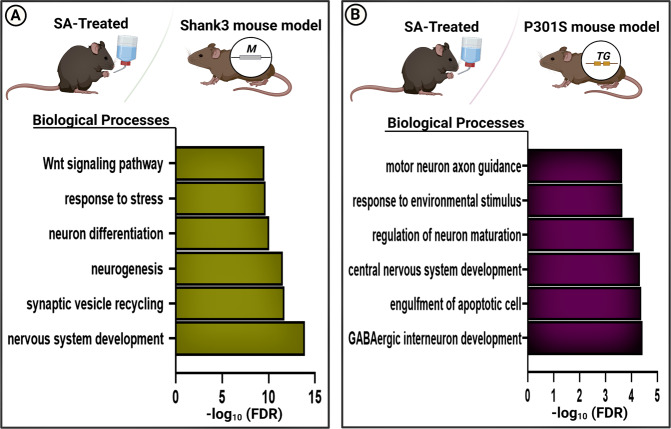
Comparative analysis between SA-treated mice and ASD/AD mouse models. BP analysis was conducted on the shared cortical SNO proteins between **A)** SA-treated group and the *Shank3* mutant group; **B)** SA-treated group and *P301S* mutant group. * Bars represent the –log10 of the Benjamini corrected false discovery rate (FDR).

Previous studies showed similarities between environmentally and genetically induced neurological disorders. In particular, resemblances between SA treatment and autism spectrum disorder (ASD) and Alzheimer’s disease (AD) pathologies are manifested in the behavioral dysfunctions. We conducted a comparative analysis of the SNO-proteome of SA-treated mice, ASD, and AD mouse models [42, 44]. First, the overlapped SNO proteins between the SA-treated mice, *Shank3* ASD mutant mice, and P301S AD mutant mice were identified (Fig. 5). The BP analysis of the shared SNO-proteins between SA-treated mice and the ASD mouse model revealed enrichment of “nervous system development” (FDR = 1.1868E-14), “synaptic vesicle recycling” (FDR = 1.8814E-12), “neuron differentiation” (FDR = 8.9563E-11), “response to stress” (FDR = 2.1834E-10), and “Wnt signaling pathway” (FDR = 2.7245E-10) (Fig. 5A). Full details of the GO analysis of SA-treated vs. ASD mice can be found in Supplementary Table 11. BP analysis of the shared proteins between SA-treated mice and AD mouse model showed enrichment of the “engulfment of the apoptotic cell” (FDR = 4.254E-05), “central nervous system development” (FDR = 4.643E-05), “cerebral cortex GABAergic interneuron differentiation” (FDR = 5.021E-05), “regulation of neuron maturation” (FDR = 8.186E-05), “cellular response to environmental stimulus” (FDR = 0.0002), and “motor neuron axon guidance” (FDR = 0.0002) (Fig. 5B). Full results of GO analysis of SA-treated *vs*. AD mice are presented in Supplementary Table 12. Heat maps analysis of the shared cortical proteins between arsenic and *Shank3* mutant group and between arsenic and *P301S* mutant group are shown in Supplementary Fig. 8. Supplementary Tables 13 and 14 show the ion intensity of the shared proteins between As and the two models.

## Discussion

We have investigated the involvement of NO in SA-induced neurotoxicity as well as the effects of SA on cognitive functions. A novel SNOTRAP-based MS method with bioinformatics analysis was employed to identify S-nitrosylated proteins in 0.1 ppm and 1 ppm SA-treated mice compared to the SA-free control group. This study revealed reprogramming of the SNO-proteome profile in both striatum and hippocampus following the SA treatment. GO analysis of SNO-proteins in SA-treated groups showed upregulation of S-nitrosylated proteins in both brain regions, which resulted in changes in various biological and functional pathways. This goes along with the previous Tannenbaum Lab study that showed that SA significantly increases Ca^2+^ influx followed by activation of nNOS which promotes protein SNO [47]. We further looked for the molecular convergence of SA exposure, an environmental factor of neurotoxicity, and the genetically modified mouse models of ASD and AD.

The presence of SA in drinking water led to S-nitrosylation of key proteins in the striatum region. Some of these proteins were found to be associated with the pathology of AD, PD, and ASD. Thus, microtubule-associated protein 1B (MAP-1B), which is responsible for cytoskeleton stabilization and axonal extension [62], was S-nitrosylated following the exposure of mice to SA. Meanwhile, SNO of this protein is found to be involved in the progression of AD [63]. Tau and stargazin, key proteins of AD pathology [64], were also S-nitrosylated in SA-treated mice. This observation implies that SA may lead to NO-related molecular alterations similar to those observed in AD and ASD pathologies [64].

S-nitrosylation of the key mitochondrial proteins following SA treatments may affect the mitochondrial energy generation process, which is one of the most common phenomena in neurological disorders [65]. Neuronal cells demand higher energy for adequate functioning and synaptic activity compared to other cell types. They are also more susceptible to mitochondrial metabolism defects [57]. Recently our group reported that SNO regulates mitochondrial function in *Shank3* mouse model of ASD [66]. In our current study, we found that among the proteins S-nitrosylated following SA treatment, there are proteins involved in the metabolic processes in mitochondria. These proteins were citrate synthase, isocitrate dehydrogenase, and malate dehydrogenase, which are critical for TCA (Krebs) cycle [67, 68]. Nakamura and Lipton have found that NO may compromise brain energy metabolism via aberrant SNO of key enzymes in the TCA cycle, including the three above-mentioned enzymes [68] that we found to be S-nitrosylated by SA. SNO of these proteins usually results in inhibition of their activity [69]. Therefore, we suggest that SA treatment impairs the mitochondria respiratory pathways leading to neuropathological consequences [70]. Defective mitochondria produce high levels of reactive nitrogen and oxygen species (RNS and ROS). Our data also revealed that SA leads to the formation of SNO of proteins involved in the cellular defense system against ROS and RNS. We found that SA caused SNO of hexokinase (a redox-sensitive glycolytic enzyme), mitochondrial creatine kinase (responsible for energy homeostasis), mitochondrial superoxide dismutase 2, peroxiredoxin, glutathione S-transferases (antioxidant enzymes), DJ1 (sensor of oxidative stress), polyubiquitin, and heat shock protein 70 (chaperon proteins). Consequently, S-nitrosylation induced by SA might be responsible for increased oxidative stress in neurons, which further leads to cell death as reported in AD and PD [71–74]. To confirm the effects of SA exposure on mitochondrial respiration, metabolism, and oxidative/nitrosative stress, additional experiments need to be carried out in the future.

BP analysis of the SNO-proteins in the hippocampus region showed the enrichment of biological processes involving cytoskeleton remodeling, which is known to be involved in a variety of neurological disorders such as PD, AD, and Huntington’s disease [75, 76].

Dysfunction of cytoskeleton remodeling is a key pathological feature in many neurological disorders [77]. Several cytoskeleton remodeling proteins appeared to be S-nitrosylated following SA treatment. One of them was profilin, an actin-binding protein, which is involved in synaptogenesis and regulates adult spine plasticity [78]. Profilin becomes inhibited under S-nitrosylation [77]. Meanwhile, reduced activity of this protein has been reported in patients with Fragile X syndrome representing the most common cause of ASD [78]. Inhibition of profilin may be responsible for aberrant regulation of adult spine plasticity. PTK7, which was also S-nitrosylated, is a receptor that regulates the polarity, movement, and migration of cells [79]. SNO-PTK7 reduces its catalytic activity [80] and may lead to different developmental disorders [79]. Many other cytoskeleton proteins, such as spectrin, myelin basic protein, beta fodrin, actinin alpha 1, F-actin, microtubule-actin cross-linking factor 1 (MACF1), plakoglobin, and others were also S-nitrosylated. We suggest that aberrant SNO of cytoskeleton proteins induced by SA may be responsible for the development of neurological disorders [77].

Our BP analysis of SNO-proteins in striatum shows the involvement of apoptotic-related processes in the SA-induced neurotoxicity. Therefore, we performed Western blot analysis to measure the protein levels of the biomarkers of apoptosis and DNA damage such cleaved poly (ADP-ribose) polymerase-1 (PARP-1) and cleaved caspase-3. PARP-1 is a nuclear protein, which plays a major role in repairing DNA damage, transcription, regulation of astrocyte and microglial function, long-term memory, and aging [81–83]. Caspases (cysteine-dependent aspartate specific proteases) are responsible for the initiation and execution of apoptosis within a cell. Cleavage of PARP-1 by caspase 3 is a hallmark of apoptosis [84–86], which has been implicated in several neurological diseases (e.g. cerebral ischemia [87], AD [88], multiple sclerosis [89], PD [90], traumatic brain injury [91], and ASD [92, 93]). AS resulted in a dose-depended increase in the levels of cleaved PARP-1 and also increased the levels of cleaved caspase-3 (Fig. 3) indicating activation of apoptosis. We suggest that the SNO of key apoptotic proteins during exposure to AS may converge into cell death. This hypothesis is supported by our previous study that shows that exposure of primary neuronal culture to AS brought about NO-mediated apoptosis manifested in the enhanced TUNEL reaction [47]. This is consistent with the data of others as well [61].

SA-induced S-nitrosylation of several other key proteins that are reported to be involved in the AD pathogenesis, such as protein kinase c (PKC) [71–74, 94], cullin-5 [95], and glyceraldehyde-3-phosphate dehydrogenase (GAPDH) [96, 97]. The S-nitrosylated GAPDH also participates in apoptotic cell death [98].

We performed a large-scale behavioral tests in mice to test whether SA-related aberrant SNO signaling leads to behavioral deficits. SA treatment resulted in some behavioral deficits similar to that of AD [99, 100] and ASD [101, 102]. We found that the SA-treated mice were not able to differentiate between familiar and new objects, which shows a defect in novelty-seeking, learning, and memory. These mice were also less interested in the novel object compared to the control group in the NOE test. These behavioral abnormalities are common to many neurological disorders including AD [99, 100] and ASD [101, 102]. We suggest that the NO and SNO-dependent abnormal alterations of the key biochemical and biological processes in the brain (discussed above) led to the behavioral deficits of SA-treated mice. To confirm this hypothesis, NO manipulations followed by behavioral examinations need to be carried out in the future.

To examine the NO-related mechanisms shared between SA and both ASD and AD mouse models and test the molecular convergence between the environmental factor, AS in drinking water, and the genetic mutations causing AD and ASD pathology, we have conducted a comparative bioinformatics analysis of the SNO-proteome. In particular, we identified biological processes enriched by S-nitrosylation that are common to SA-treated and the *Shank3* mutant mice. We found processes such as “neurogenesis”, “Wnt signaling pathway” [103] and “nervous system development”, which are known to be involved in neurodevelopmental and behavioral abnormalities [104, 105]. BP analysis also revealed that SNO enrichment of “motor neuron axon guidance,” “engulfment of the apoptotic cell,” and “response to environmental stimulus,” were common to both SA-treated mice and the *P301S* transgenic mouse model of AD. This is consistent with the previous works [106–108] that point to the involvement of these processes in neurodegeneration and cell death. Collectively, the results of this study imply that both the environmental (AS) and genetic factors (*Shank3* and *P301S* mutations) may lead to aberrant SNO signaling affecting common biological processes and pathways that might contribute to neuropathology.

In conclusion, this multidisciplinary study explores for the first time the effects of low doses of AS on SNO signaling in the striatum and hippocampus. It also examines the AS effects on the biological processes involved in neuropathology and behavioral deficits. AS induced S-nitrosylation of key proteins implicated in mitochondrial respiratory function, endogenous antioxidant systems, transcriptional regulation, cytoskeleton maintenance, and regulation of apoptosis. Dysfunction of these proteins caused by aberrant S-nitrosylation results in the impairment of neurodevelopmental processes, neuronal functions, and neuronal cell viability, which are features that also observed in ASD and AD pathology. Unsurprisingly, exposure of mice to SA in drinking water led to some but not all behavioral deficits, which are known in ASD and AD pathologies. In some studies, NOS inhibitors are found to be protective in neurodegenerative disorders such as PD [109, 110], AD [111, 112], cerebral hypoxia-ischemia [113], and in other toxic insults [113]. NOS inhibitors are also reported to be an effective antidepressants [114]. Here we suggest that the use of NOS inhibitors can be an effective therapeutic strategy against NO-mediated neurotoxicity. Further investigation of these mechanisms is likely to reveal new potential drug targets for the treatment of AS-mediated neurotoxicity.

## Supplementary information

SUPPLEMENTAL MATERIAL

Supp. Figure 1

Supp. Figure 2

Supp. Figure 3

Supp. Figure 4

Supp. Figure 5

Supp. Figure 6

Supp. Figure 7

Supp. Figure 8

Supp. Table 1

Supp. Table 2

Supp. Table 3

Supp. Table 4

Supp. Table 5

Supp. Table 6

Supp. Table 7

Supp. Table 8

Supp. Table 9

Supp. Table 10

Supp. Table 11

Supp. Table 12

Supp. Table 13

Supp. Table 14
