# Supplementary material for: Arsenic alters nitric oxide signaling similar to autism spectrum disorder and Alzheimer’s disease-associated mutations
Source: Transl Psychiatry. 2022 Mar 28;12:127. doi: 10.1038/s41398-022-01890-5 (PMC8964747; doi:10.1038/s41398-022-01890-5)
Supplement: Supplementary file 1 — SUPPLEMENTAL MATERIAL [file 41398_2022_1890_MOESM1_ESM.docx]

**Arsenic alters nitric oxide signaling similar to autism spectrum disorder and Alzheimer's disease-associated mutations**

Manish Kumar Tripathi^1^, Maryam Kartawy^1^, Shelly Ginzburg^1^, and Haitham Amal^1,#^

1 Institute for Drug Research, School of Pharmacy, Faculty of Medicine, The Hebrew University of Jerusalem, Jerusalem, Israel

# Corresponding Author: Prof. Haitham Amal, Email: Haitham.amal@mail.huji.ac.il

Supplementary Tables are uploaded as Excel files:

Table 1: IDs of SNO-proteins in the striatum in all different groups.

Table 2: IDs of SNO-proteins in the hippocampus in all different groups.

Table 3: System biology analysis in the striatum of the control group

Table 4: System biology analysis in the striatum of 0.1 ppm SA-treatment group

Table 5: System biology analysis in the striatum of 1 ppm SA-treatment group

Table 6: System biology analysis in the hippocampus of the control group

Table 7: System biology analysis in the hippocampus of 0.1 ppm SA-treatment group

Table 8: System biology analysis in the hippocampus of 1 ppm SA-treatment group

Table 9: Pathway analysis in the striatum of SA treated groups

Table 10: Pathway analysis in the hippocampus of SA treated groups

Table 11: System biology analysis of the shared cortical SNO proteins between SA-treated group and *Shank3* mutant group

Table 12: System biology analysis of the shared cortical SNO proteins between SA-treated group and P301S mutant group

Table 13: Relative ion intensity of shared cortical SNO proteins between SA-treated group and Shank3 mutant group

Table 14: Relative ion intensity of shared cortical SNO proteins between SA-treated group and P301S mutant group

Supplementary figures

Supp. Fig. 1

**Figure to legends -** GO analysis was conducted on the SNO-Proteins of striatum and hippocampus. (A) MF analysis in all groups of striatum. (B) CC analysis in all groups of striatum (C) CC analysis in all groups of hippocampus. Each bar represents the −log10 of the False discovery rate (FDR) value.

**Supp. Fig. 2**

**Figure to legends -** Protein Protein Interactions network analysis of the SNO proteins that were found exclusively in 1ppm SA treated group A) SNO proteins in Hippocampus, B) SNO proteins in striatum.

**Supp. Fig. 3**

**Figure to legends -** Pathway analysis revealed enrichment of “Neurophysiological process-Synaptic vesicle fusion and recycling in nerve terminals” in Striatum of SA treatment group. Red circles show the proteins enriched in striatum of 1 ppm SA group and green circles showed the protein enriched in both 0.1 ppm and 1 ppm treated groups.

**Supp. Fig. S4**

**Figure to legends -** Pathway analysis revealed enrichment of “Signal transduction_mTORC2 downstream signaling” in hippocampus SA treated group. Red circles shows the proteins enriched in hippocampus of 1 ppm SA group and black circles shows the proteins enriched in both 0.1 ppm and 1 ppm treated groups.

**Supp. Fig. S5**

**Figure to legends -** Quantitative analysis of the SNO-proteins in Striatum. A) Volcano Plot analysis was conducted on the shared SNO proteins in the control vs. 0.1 ppm treated group. (B) Heat map analysis representing the differential relative abundance of the shared SNO-proteins in control vs. 0.1 ppm. The relative abundance scale was normalized by –log10.

**Supp. Fig. S6**

**Figure to legends -** Volcano plot analysis of the SNO proteins in the hippocampus was conducted on A) the shared proteins in the control vs. 1 ppm SA group and B) the shared proteins in the control vs. 0.1 ppm group.

**Supp. Fig. S7**

**Figure to legends -** (A) Representative western blot of striatal homogenate from control, 0.1 ppm SA and 1 ppm SA treated group shows no significant difference in the level of LC3B (molecular weight -17 kDa), P-62 (molecular weight-62 kDa) and p-m-TOR (molecular weight-289 kDa) in striatum of 0.1 ppm and 1 ppm SA treated group as compared with control group. Immunoblotting analysis of (B) LC3B, (C) P-62 and (D) p-m-TOR in all groups of striatum. The data are presented as mean values ± SEM (n = 5). Two tailed t-test was conducted.

**Supp. Fig. S8**

**Figure to legends -** (A) Heat map analysis representing the differential relative abundance of the shared SNO-proteins in Arsenic vs. *Shank3* mutant group. The relative abundance scale was normalized by –log10. (B) Heat map analysis representing the differential relative abundance of the shared SNO-proteins in Arsenic vs P301S mutant group. The relative abundance scale was normalized by –log10.
