# Supplementary figures and images for: Arsenic alters nitric oxide signaling similar to autism spectrum disorder and Alzheimer’s disease-associated mutations
Source: Transl Psychiatry. 2022 Mar 28;12:127. doi: 10.1038/s41398-022-01890-5 (PMC8964747; doi:10.1038/s41398-022-01890-5)

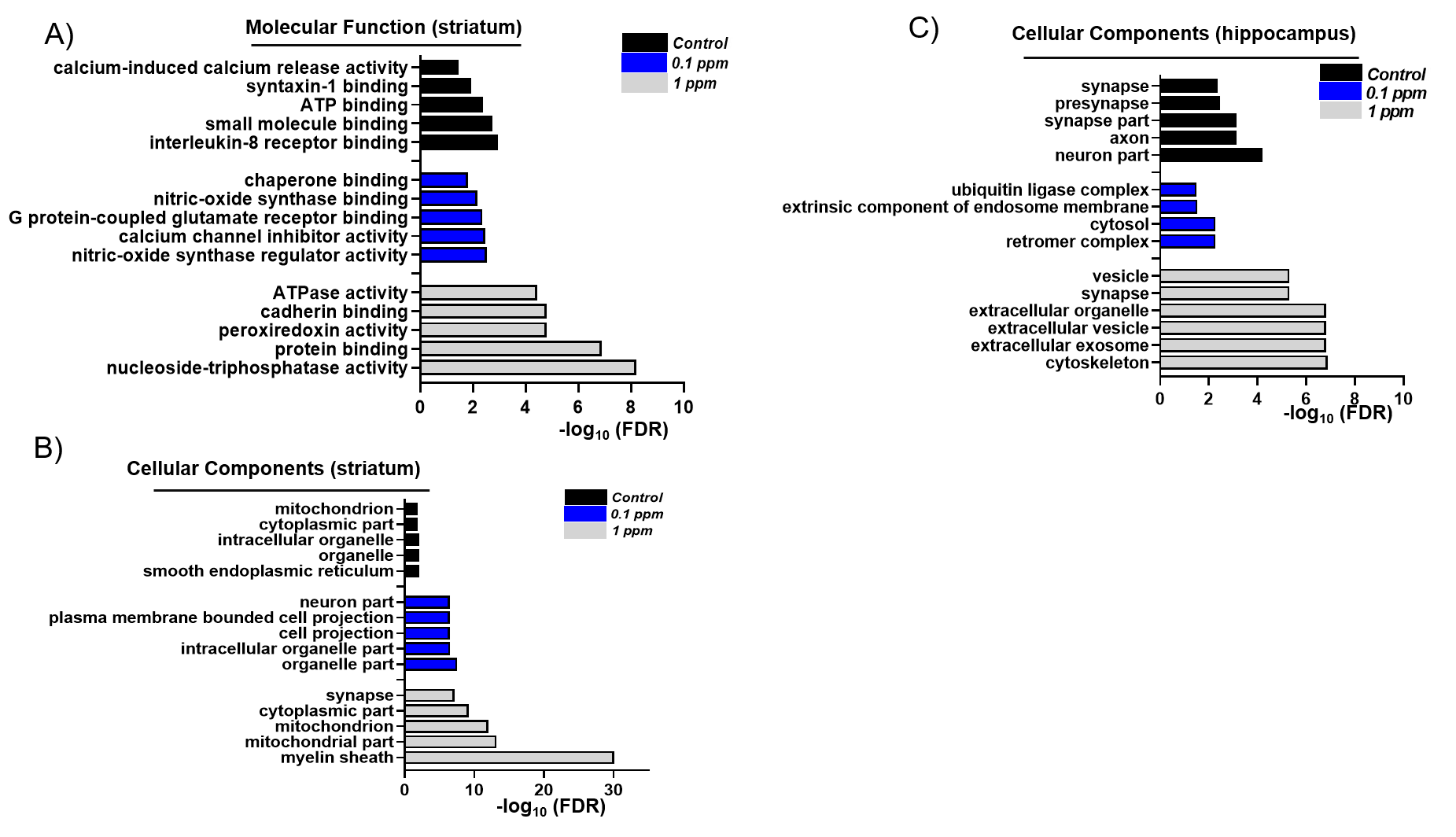

Supplement: Supplementary file 2 — Supp. Figure 1 [file 41398_2022_1890_MOESM2_ESM.png]

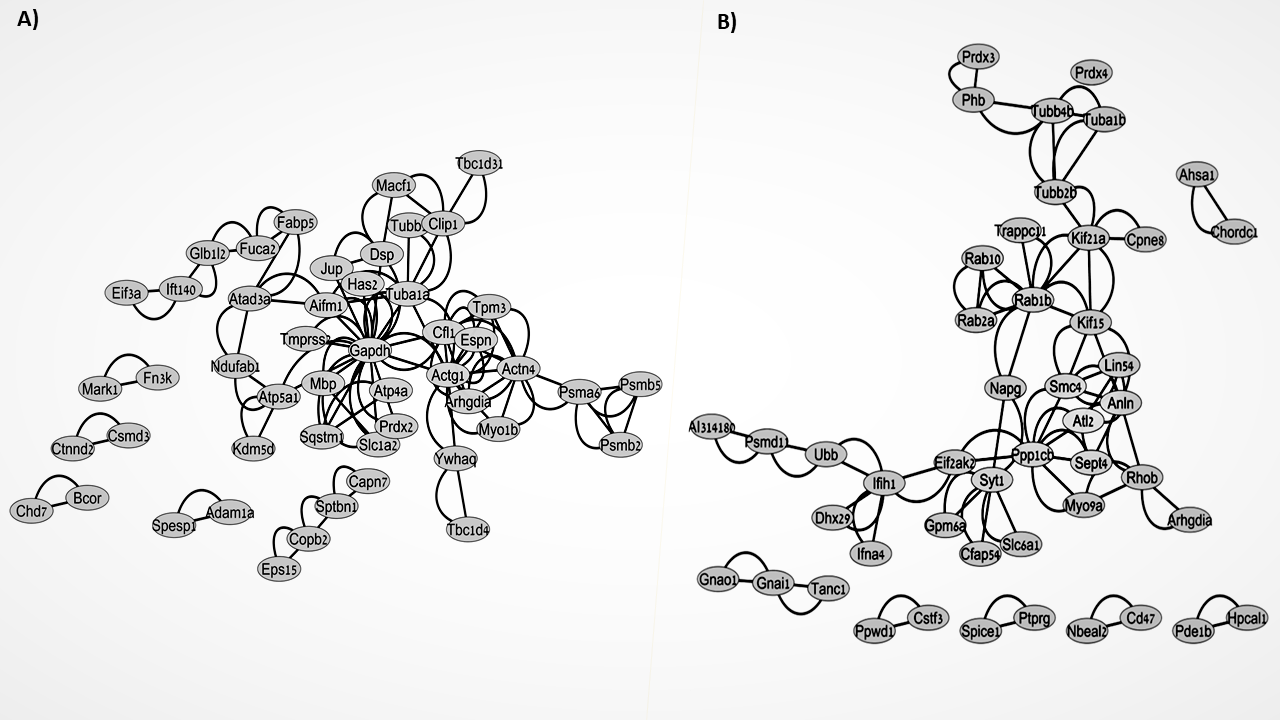

Supplement: Supplementary file 3 — Supp. Figure 2 [file 41398_2022_1890_MOESM3_ESM.tif]

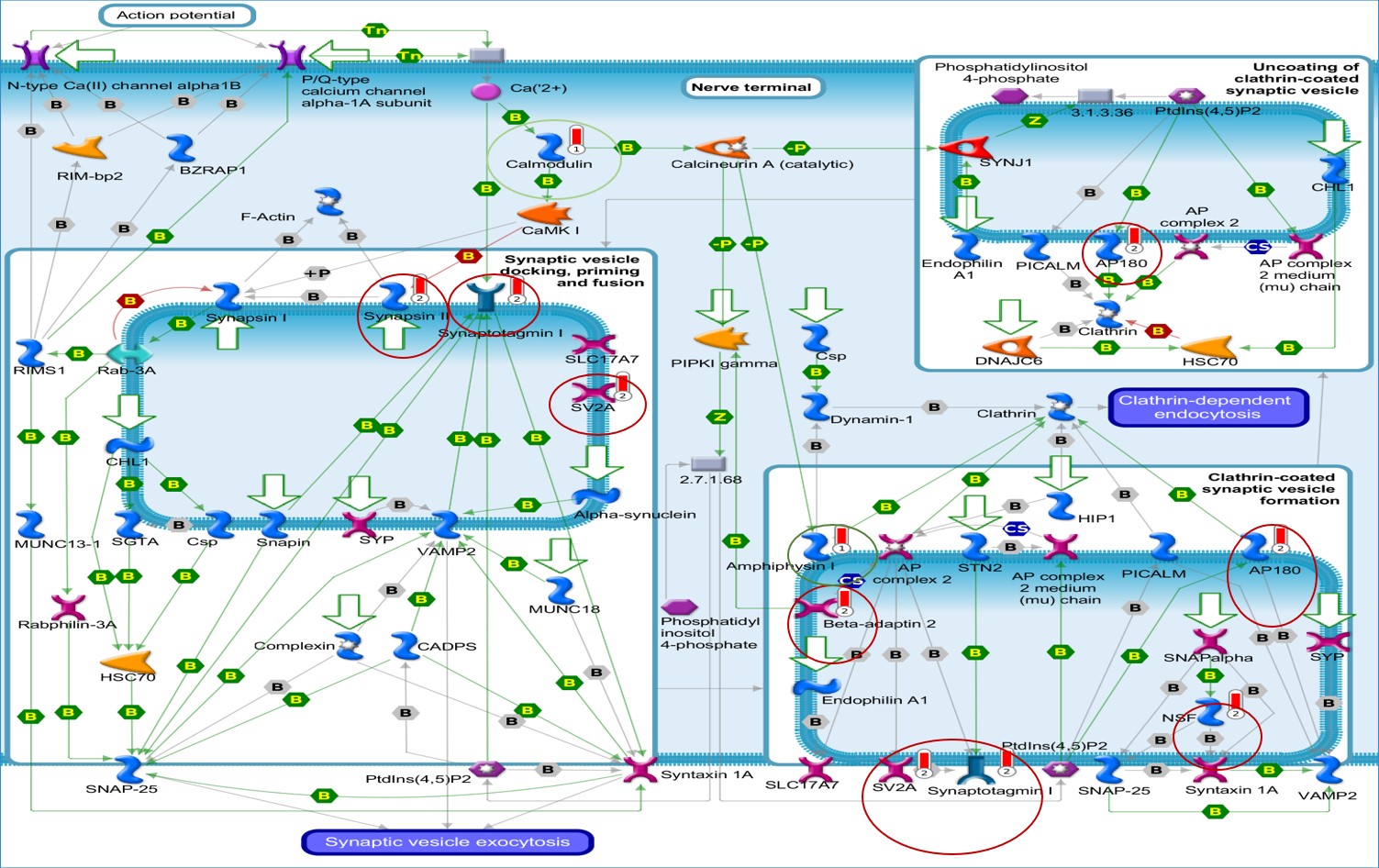

Supplement: Supplementary file 4 — Supp. Figure 3 [file 41398_2022_1890_MOESM4_ESM.jpg]

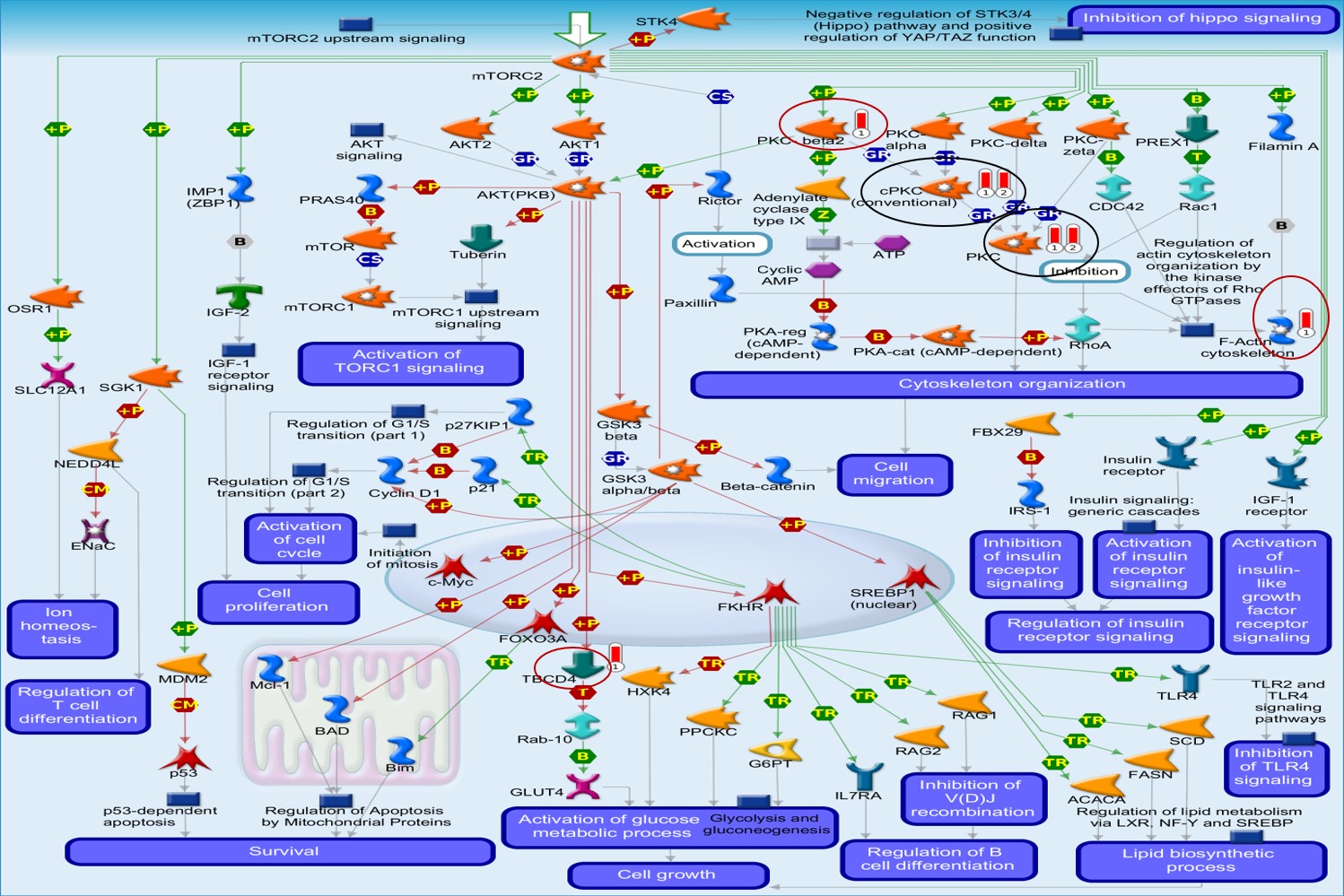

Supplement: Supplementary file 5 — Supp. Figure 4 [file 41398_2022_1890_MOESM5_ESM.jpg]

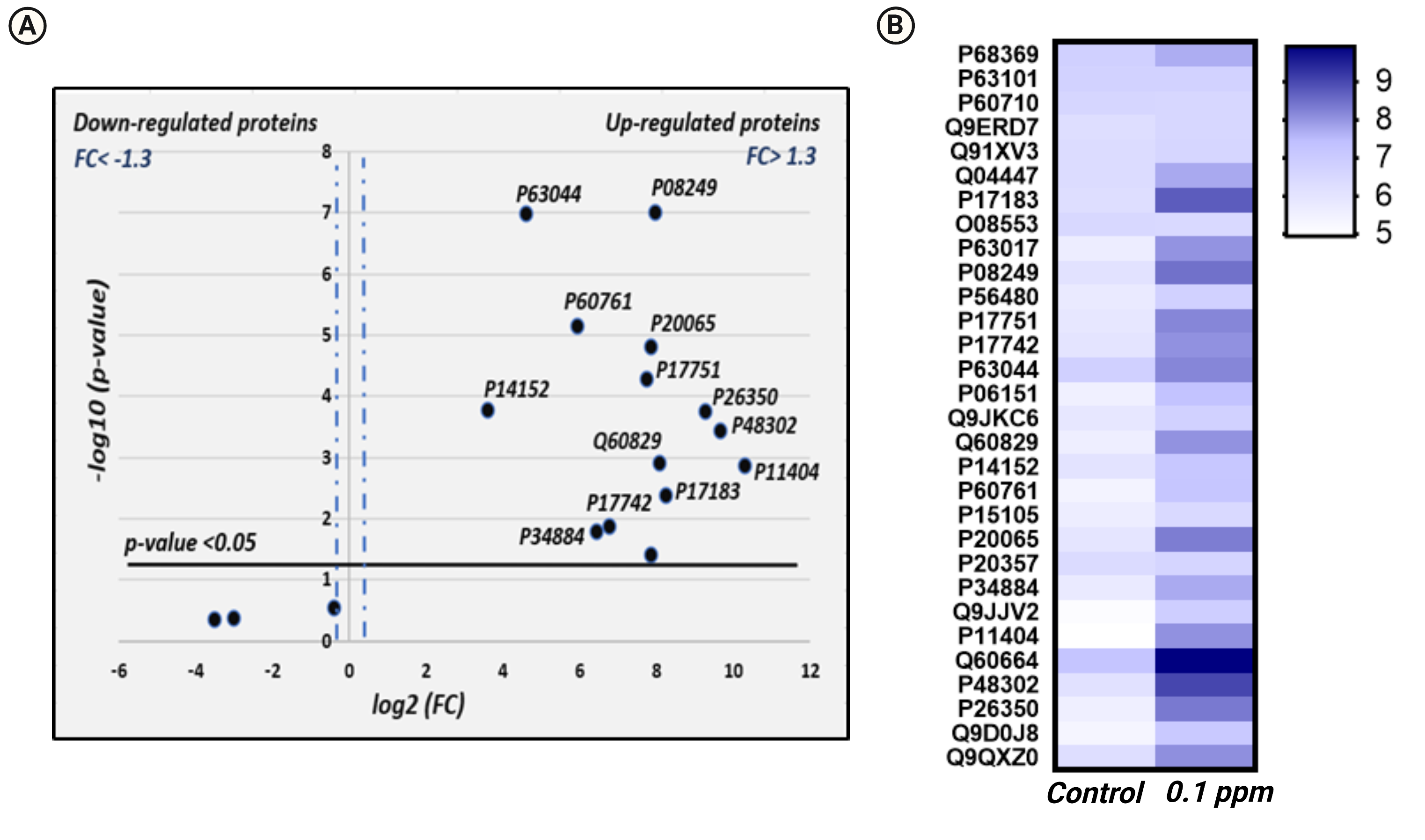

Supplement: Supplementary file 6 — Supp. Figure 5 [file 41398_2022_1890_MOESM6_ESM.png]

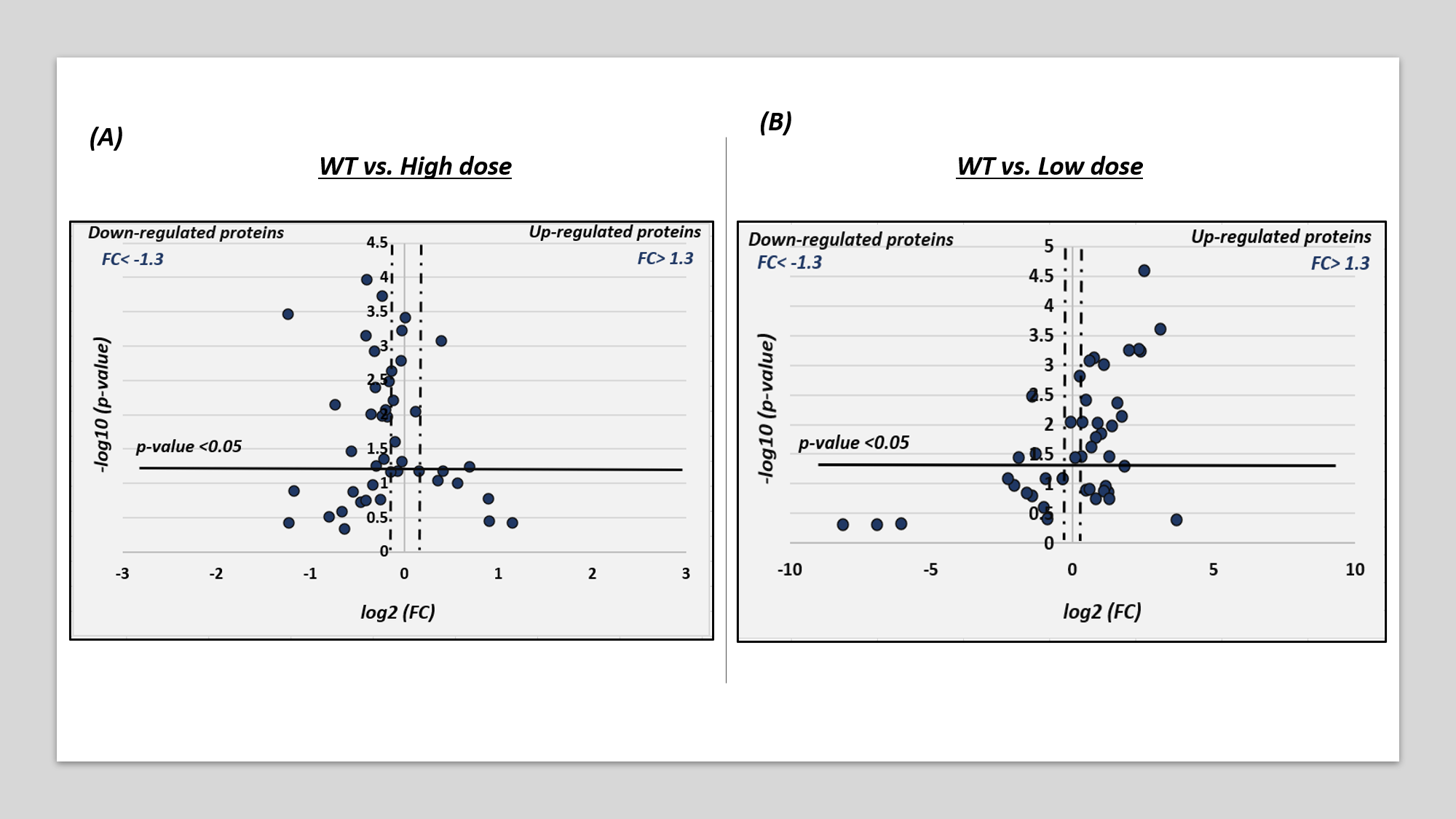

Supplement: Supplementary file 7 — Supp. Figure 6 [file 41398_2022_1890_MOESM7_ESM.png]

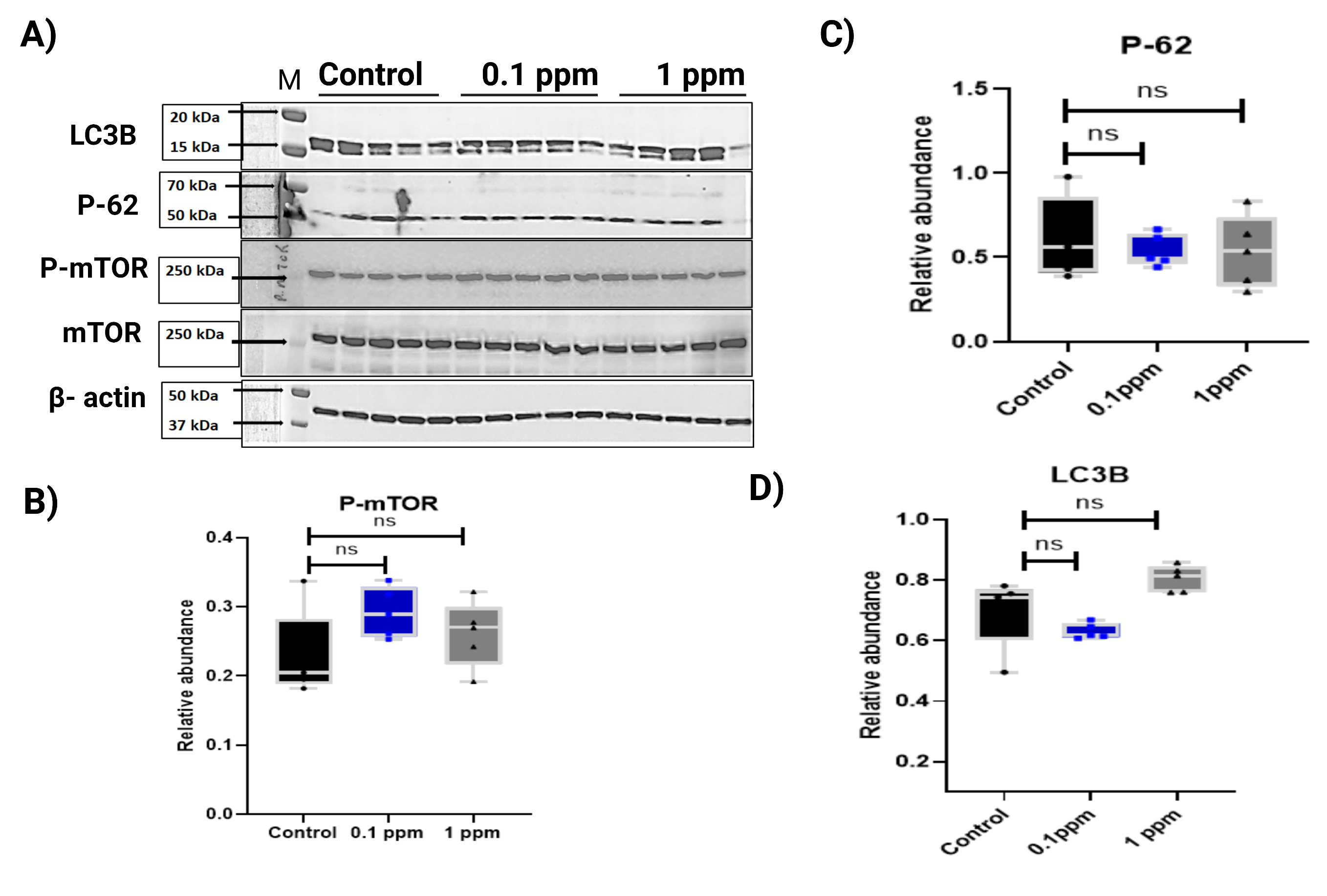

Supplement: Supplementary file 8 — Supp. Figure 7 [file 41398_2022_1890_MOESM8_ESM.png]

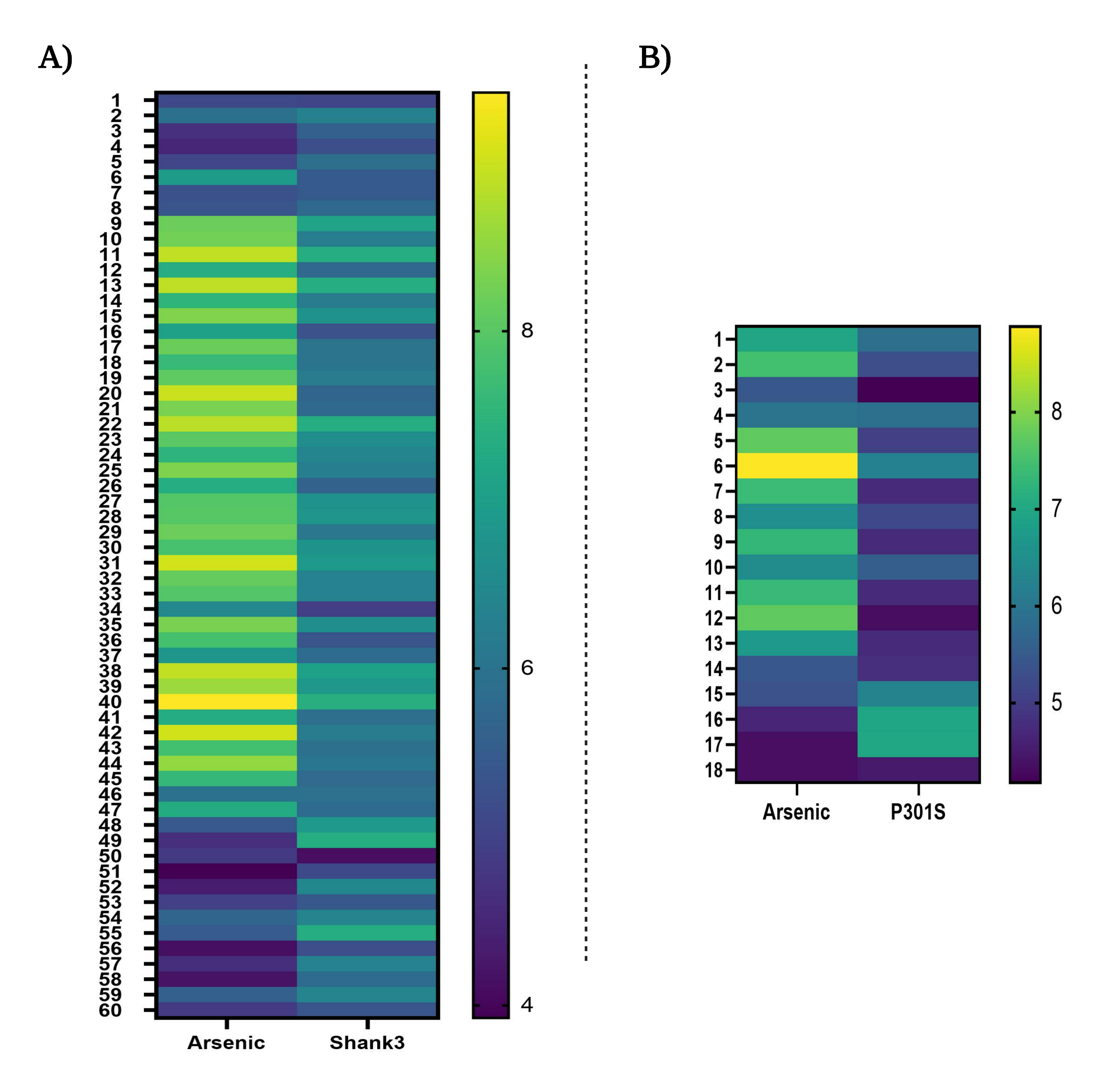

Supplement: Supplementary file 9 — Supp. Figure 8 [file 41398_2022_1890_MOESM9_ESM.png]
